# Management Challenges of Deep Infiltrating Endometriosis

**DOI:** 10.22074/IJFS.2020.134689

**Published:** 2021-03-11

**Authors:** Maurizio Nicola D’Alterio, Gianmarco D’Ancona, Mohamed Raslan, Raffaele Tinelli, Angelos Daniilidis, Stefano Angioni

**Affiliations:** 1 Department of Surgical Sciences, University of Cagliari, Cagliari, Italy; 2 Department of Obstetrics and Gynaecology, Tanta University, Tanta, Egypt; 3 Department of Obstetrics and Gynaecology, ‘Valle d’Itria’ Hospital, Martina Franca, Taranto, Italy; 4 Department of Obstetrics and Gynaecology, 2nd University Clinic of Obstetrics and Gynaecology, Aristotele University of Thessaloniki, Thessaloniki, Greece

**Keywords:** Endometriosis, Surgery, Therapy

## Abstract

Deep infiltrating endometriosis (DIE) is the most aggressive of the three phenotypes that constitute endometriosis. It can affect the whole pelvis, subverting the anatomy and functionality of vital organs, with an important negative impact on the patient’s quality of life. The diagnosis of DIE is based on clinical and physical examination, instrumen- tal examination, and, if surgery is needed, the identification and biopsy of lesions. The choice of the best therapeutic approach for women with DIE is often challenging. Therapeutic options include medical and surgical treatment, and the decision should be dictated by the patient’s medical history, disease stage, symptom severity, and personal choice. Medical therapy can control the symptoms and stop the development of pathology, keeping in mind the side effects derived from a long-term treatment and the risk of recurrence once suspended. Surgical treatment should be proposed only when it is strictly necessary (failed hormone therapy, contraindications to hormone treatment, severity of symp- toms, infertility), preferring, whenever possible, a conservative approach performed by a multidisciplinary team. All therapeutic possibilities have to be explained by the physicians in order to help the patients to make the right choice and minimize the impact of the disease on their lives.

## Introduction

Endometriosis is a chronic inflammatory disease caused by the presence of ectopic endometrial tissue, which reacts to changes in the ovarian steroids, oestrogen and progesterone as expressed by proliferation, differentiation, and bleeding (1).

Estimating the exact prevalence of endometriosis is a challenge since many women with this pathology are asymptomatic, while others may report non-specific symptoms. It mostly occurs in women of reproductive age with a prevalence of 7-10% and 50% of women with subfertility (2, 3), and is one of the most frequent chronic gynaecological diseases that often affects quality of life and fertility (4, 5). 

Endometriosis can take one of three forms, depending on the clinical presentation and management: peritoneal or superficial endometriosis, ovarian endometrioma (OMA), or deep infiltrating endometriosis (DIE). DIE is the most aggressive form, which affects 20% of women who suffer from endometriosis (6).

At present, there is no clear agreement on the definition of DIE. Many authors define DIE as the presence of endometriotic lesions over 5 mm in depth under the peritoneal surface; others define it as a pathologic entity, which is called “adenomyosis externa”. The 5 mm definition allows the understanding of lightly deeper classic lesions (type I). It would be more suitable to define DIE as adenomyosis externa with unique lesions (infrequently two or three) that are large (mainly >1 cm in diameter), and are reported as type II and type III lesions (7). According to a recent Cochrane meta-analysis, DIE is also defined as the infiltration of fibrous and muscular tissue in organs and anatomic structures affected by endometriosis, including endometrial tissue, with no reference to the extent of lesion depth underneath the peritoneum (8).

Recent literature have shown that many factors contribute to the growth and development of endometriosis: genetic, hormonal, immunological factors play a role, and even intestinal permeability may be involved (9-12). In the absence of other types of endometriosis, the isolated presence of DIE was only observed in 6.5% of cases. Although it may be considered a separate entity, they all may share similar pathogenic pathways (13). To explain the pathogenesis of DIE, the Sampson’s theory has some limitations, such as the fact that endometriosis is found in only 10% of cases but the physiological process of retrograde menstruation occurs in 90% of women, or the occurrence of the endometriosis in men. Instead, the pathophysiology of DIE may be explained by the role of endometrial stem/progenitor cells and coelomic epithelial and mesenchymal cells, which could be the origin of premenarcheal pelvic endometriosis. The onset of DIE in adulthood indicates that DIE could be a retarded stage of endometriosis (14). On the other hand, there is the hypothesis that the endometriotic cells undergo tumour-like genetic and epigenetic modifications, and these changes influence the progression to DIE (15). This theory could explain the existence of the three described phenotypes of endometriosis since they could be based on different genetic mutations (11). The more intense aggressiveness of DIE compared with the other forms seems to be attributable to two main mechanisms: decreased apoptosis of endometrial cells involved in lesion sites and higher proliferation activity of those cells in response to the oxidative stress generated in these lesions (16). Furthermore, DIE is characterized by higher expression of invasive mechanisms (caused by matrix metalloproteinases and activins) and of neuroangiogenesis genes (nerve growth factor, vascular endothelial growth factor) compared with superficial and ovarian endometriosis (17).

**Fig.1:**
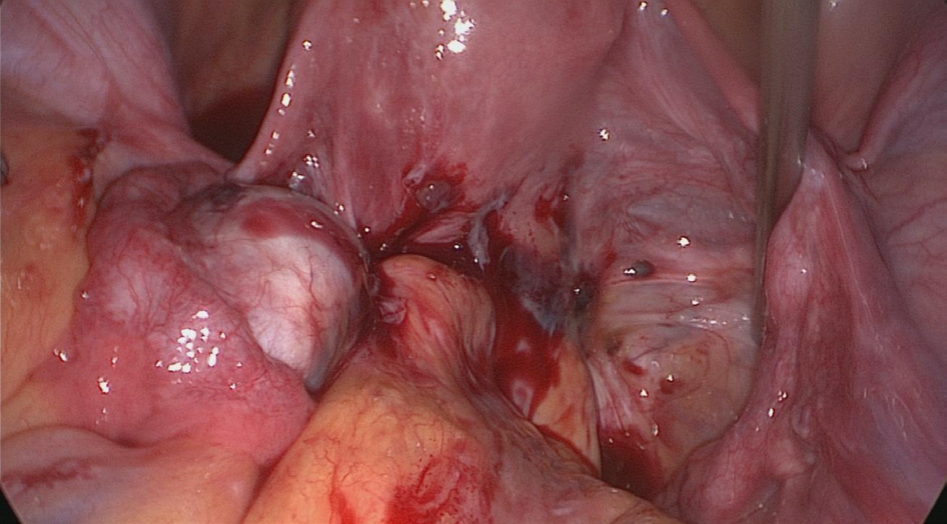
Laparoscopic view of posterior compartment deep infiltrating en- dometriosis (DIE).

DIE lesions appear to expand as benign tumours, preferentially in the pouch of Douglas, with expansion to the uterosacral ligaments, torus uterinum, cardinal ligament with uterine artery involvement, ureters, or bladder, with a preferential invasion into the anterior rectal wall [Fig.1, 18)]. 

Associated symptoms generally are related to the localizations [Table 1 (19)].

The diagnosis of DIE and, more generally, endometriosis, is based on clinical and physical examination, instrumental examination [ultrasound, magnetic resonance imaging (MRI), double-contrast barium enema (DCBE), cystoscopy, computed tomography (CT) scan], and, if surgery is needed, the identification and biopsy of lesions. With regard to clinical diagnosis, it is often difficult to obtain in asymptomatic patients or when there is an inadequate correlation between the severity of the endometriotic lesions and the intensity of the symptoms (20, 21). 

**Table 1:** Main localizations and associated symptoms of deep infiltrating endometriosis (DIE)


Localization	Symptoms

Uterosacral and cardinal ligaments, pouch of douglas, posterior vaginal fornix	Dyspareunia, dysmenorrhea, chronic pelvic pain, pelvic tenderness
Bladder, bladder-uterine septum	Urinary symptoms (frequency, urgency, dysuria, haematuria)
Ureter	Asymptomatic, colicky flank pain, haematuria
Bowel and rectovaginal septum	Dyschezia, diarrhoea, constipation, intestinal cramping, painful defecation, abdominal bloating


### Therapeutic management

The choice of the best therapeutic approach for women with DIE is often challenging. Therapeutic options include medical and surgical treatment, and the decision should be dictated by the patient’s medical history, disease stage, symptoms, severity, pregnancy desire, and personal choice (22).

### Medical treatment

Medical therapy has proved to be useful in both stopping the growth of lesions and inducing their regression, with a consequent improvement of symptoms. In addition, pharmacotherapy plays an important role in supporting surgical therapy, either in the period immediately preceding or, even more, after surgery (23). Currently available treatments include progestogens, combined oral contraceptives (COCs), danazol, gonadotropin-releasing hormone (GnRH) analogues and aromatase inhibitors (AI) (Table 2). An adequate lifestyle, a diet rich in vegetables and omega-3 polyunsaturated fatty acids, and a parallel reduction of red meat, coffee, and alcohol consumption might be important in endorsing and amplifying the benefits of medical therapy (24). In addition, promising results have come from the use of substances that act on mastocyte function and inflammation, especially in women who cannot use hormone therapy or who seek to become pregnant (25).

### Progestogens and combined oral contraceptives

Overall, progestogens and COCs are proven to be particularly effective in managing the symptoms of patients with DIE.

Norethisterone acetate (NETA) and dienogest have the best data in terms of their effects on DIE. A pilot study by Ferrero et al. (26) proved the effectiveness of NETA (5 mg/day) in improving intestinal symptoms and reducing the volume of the endometriotic nodules of 40 patients with colorectal endometriosis and stenosis of the lumen of the bowel to <60%. At the end of the trial, 60% of patients stated their satisfaction with this therapy

**Table 2:** Different therapies for the medical treatment of deep infiltrating endometriosis (DIE)


Therapy	Available forms	Advantages	Disadvantages

Progestogens and combined oral contraceptives (COCs)	Oral, intramuscular or subcutaneous injection, intrauterine devices, transdermal patches, vaginal rings	Effectively relieve DIE-associated symptomsLong-term safety Oral administration	Side effects: Abnormal uterine bleeding, nausea, breast tenderness, fluid retention, mood changes, risk of venous thromboembolismNeed for chronic administration due to rapid return of pain after treatment discontinuation
Gonadotropin-releasing hormone (GnRH) analogues	Most common administration route is intramuscular Oral administration: GnRHant (Elagolix)	Effective in the relief of DIE-associated symptoms Remarkable results when administered pre- or post-surgery, even on digestive symptoms	Require hormone add-back therapy due to adverse effects (menopausal symptoms, bone mineral density loss) Cannot be prolonged beyond six months because of the likelihood of hypoestrogenism? Early recurrence of symptoms after treatment suspension
Danazol	Most common administration route is vaginal	Effective in the relief of DIE-associated symptoms Well-tolerated	Side effects due to hyperandrogenism (acne, hirsutism)No contraceptive function
Aromatase inhibitors (AI)	Oral administration	Inhibits only local oestrogen production in endometriotic implantsPromising effect for managing severe endometriosis-associated pain Oral administration	Not yet approved for use in clinical practice for endometriosis Not effective if not associated with other drugs that inhibit ovulation


Dienogest entered the market as a drug dedicated to the treatment of endometriosis; many studies suggested its effectiveness in the management of rectovaginal or bowel endometriosis. Leonardo-Pinto et al. (27) prescribed dienogest (2 mg/day for 12 months) for 30 women who were dissatisfied with their previous progestogen therapy. Participants reported a significant reduction in intestinal pain. However, the authors did not notice any decrease in bowel lesion size. Yela et al. (28) reported improved symptoms, such as defecation pain, from the second month of therapy. After six months of therapy with dienogest (2 mg/day), they noted a reduction in the mean volume of the bowel endometriotic nodules. Moreover, with the same dosage of dienogest, Angioni et al. (29, 30) observed an improvement in symptoms and reduced nodules size in patients affected by bladder DIE. Similar results for symptoms and cyst volume were obtained in patients with endometrioma, which suggested that the absence of endometriosis/endometrial bleeding could be a key mechanism in these results.

COCs, by decreasing the nerve fibre density in DIE lesions, enhancing apoptosis, and regulating cell apoptosis in endometriotic cells, demonstrated optimistic results (31). Since COCs supply a higher doses of oestrogen than what occurs physiologically, the rationale for their use has been questioned because their dose may stimulate endometriosis (32). Moreover, COCs may have additional side effects and contraindications compared with progestins. Therefore, European Society of Human Reproduction and Embryology (ESHRE) guidelines recommend progestins as a first-line medical therapy (33).

### Gonadotropin-releasing hormone analogues

GnRH agonists (GnRHa) play an important role in the treatment of endometriosis. Their effect on DIE has mainly been documented with remarkable results. Fedele et al. (34) evaluated the effect of these drugs (leuprolide acetate depot, 3.75 mg, one ampoule intramuscularly every 28 days for six months) in patients with symptomatic rectovaginal nodules. Many of the patients described improvement in their symptomatology during six months of treatment, but 85% of these patients required a new therapy cycle during the same year for an early recurrence of symptoms. Roman et al. (35), in a study of patients with rectal endometriosis, reported that Triptorelin (11.25 mg) plus one daily dose of percutaneous oestradiol (0.1%) had the same effectiveness in bowel endometriosis when administered three months before surgery to control digestive disorders and when prescribed after surgery in case of incomplete resection of the rectum DIE. Triptorelin acetate (3.75 mg, monthly intramuscular injection for six months) was evaluated by Angioni et al. (36) as a post-surgical medical treatment in patients with rectovaginal DIE. The outcomes of this research showed an improvement of symptoms in those patients in whom total eradication of the pathology was not feasible.

The GnRH antagonist (GnRHant), Elagolix, is another drug that is proving to be effective in the management of DIE. This drug has some advantages in comparison with GnRHa because of its oral formulation, rapid elimination from the body due to its short half-life, and a lower incidence of adverse events (37).

### Danazol and aromatase inhibitors

Danazol, a 17 alpha-ethinyl testosterone derivative, operates principally by suppressing the luteinizing hormone (LH) wave and steroidogenesis. It has been shown to have similar pain control to GnRH-agonists. However, its hyperandrogenic side effects such as hirsutism, acne, weight gain, and deepening of the voice are common (38). At present, the most common administration route for danazol is vaginal (vaginal ring, gel, or capsule) in order to reduce systemic side effects. A prospective study conducted on 21 patients evaluated the effect of long-term treatment with a low dose of vaginal danazol (200 mg/day) for 12 months on DIE. The results demonstrated an improvement in pain within three months of treatment, with total resolution by six months, and the effect remained over the 12 months of treatment, associated with a volume reduction of rectovaginal nodules (39).

AIs inhibit the secretion of local oestrogen in endometriosis implants and, while they are not recommended for endometriosis therapy, many studies have examined their use in DIE pain management. In combination treatment with COCs, progestogens and GnRH analogues, AIs are a therapeutic choice typically reserved for the management of severe endometriosis-associated pain. Increased follicle-stimulating hormone (FSH) levels and successive superovulation would be induced by monotherapy with AIs offered to reproductive-age women, which culminate in ovarian cyst production due to the resultant increase in FSH. For this effect, AIs are associated with FSH-suppression drugs such as COCs, progestogens, or GnRHa (40). In an open-label prospective randomized study, Ferrero et al. (41) evaluated the efficacy and tolerability of letrozole (2.5 mg/day) combined with NETA (2.5 mg/ day) or Triptorelin (11.25 mg for three months) in the treatment of pain produced by rectovaginal endometriosis for six months. During therapy, chronic pelvic pain and profound dyspareunia decreased considerably in both groups with no substantial variation between the groups. The reduction in the volume of endometriotic nodules was significantly higher in the Triptorelin group, where, 77.8% of women reported adverse reactions that included menopause symptoms and loss of bone mineral density. This study did not show indications that AIs may function because, when hormonal drugs are combined (letrozole plus NETA), the particular effect of each compound cannot be discriminated. On the other hand, AIs are ineffective unless they are combined with other medications that prevent ovulation. Due to a lack of data on the use of AIs for the treatment of patients with endometriosis and, in particular DIE, their use should be considered experimental. It should be considered only when patients are refractory to common hormonal or surgical therapy and in the context of a clinical study (42).

### Other medical treatments

Selective progesterone receptor modulators (SPRMs) can have shifting impacts on progesterone receptors in different tissues, ranging from being a pure agonist or mixed agonist/antagonist or a pure antagonist. Through their pro-apoptotic effects, anti-inflammatory effects (decreasing cyclooxygenase-2 expression) and reducing cell proliferation, as demonstrated by a decrease in Ki-67 expression, they can play a role to regression and atrophy of endometriotic lesions in mice. 

In terms of selective oestrogen receptor modulators (SERMs), by reducing the proliferation of cell nuclear antigen and the expression of oestrogen receptor in the endometrium, promising results were reported in endometriosis treatment with the use of Bazedoxifene (BZA) in a mice model (43). Nevertheless, the effectiveness of both SPRMs and SERMs for endometriosis management have yet to be established in humans. In light of the most recent discoveries, some angiogenic and proinflammatory factors may have key roles in the pathogenesis of endometriosis. Therefore, drugs, such as anti-TNF-alpha, cyclooxygenase-2 inhibitors, growth factor inhibitors, and endogenous angiogenesis inhibitors have been tested for endometriosis treatment. However, there is still a lack of clinical evidence of the efficacy and safety for most of these drugs (42).

### Surgical treatment

Surgical treatment of DIE is indicated in patients who do not respond to medical therapy and have significantly severe symptoms (e.g., hydronephrosis caused by ureteral stenosis or intestinal obstruction). The goal is complete eradication of this pathology and the achievement of good long-term outcomes in terms of pain relief and recurrence rates, while trying to respect the functional anatomy of the involved organs. Because of the complexity of surgery, a multidisciplinary approach that involves colorectal surgeons and urologists is often essential to reduce the risk of complications and the hospital stay (44).

### Rectovaginal and bowel endometriosis

During surgery for rectovaginal and bowel endometriosis, the surgeons can use a nerve-sparing laparoscopic technique to support urinary and bowel function, which allows for conserving the inferior hypogastric nerve plexus and identifying all of the anatomic structures in the posterior and lateral parametrium prior to removing the endometriotic lesions (45). A prospective study that compared a patients who underwent the nerve-sparing procedure and those treated with classical resection showed shorter mean time of self-catheterization of the catheter (40 days versus 121 days, respectively) and less severe bladder, rectal, and sexual dysfunctions (46). Another study by Angioni et al. (47) demonstrated that laparoscopic radical excision of DIE with excision of the posterior vaginal fornix might be the best approach in terms of long-term well-being, even if the vagina is apparently disease-free.

Most rectovaginal septum lesions arise from the posterior vaginal fornix and subsequently infiltrate the anterior rectal wall. The surgical approach for this kind of lesion can be conservative and include nodulectomy and shaving of the lesion, discoid excision, or, in selected cases, radical surgery where the involved intestinal tract is resected. Small/mid-rectal nodules that only infiltrate the muscular layer and are free of advanced stenosis of the rectal lumen can be completely removed without opening the bowel. The main advantage of rectal shaving is the ability to treat a bowel infiltration without the need to open and suture the rectal wall (48, 49). Complications include accidental intestinal perforation (2%), rectovaginal fistula (0.24%), intraoperative haemorrhage (0.08%), and catheterization for a maximum duration of six weeks (0.19%) (50). Roman et al. (51) stated that this technique has a more beneficial impact on postoperative intestinal function compared to intestinal resection. As regards the risk of recurrence of symptoms and lesions after this procedure, most publications describe recurrence of symptoms and lesions in <10% of cases. Conversely, according to Meuleman et al., the shaving technique should be reserved for superficial lesions, that is, those that do not cross the muscular layer (52).

An alternative closed technique has been suggested for cases of small anterior rectal wall small nodules localized up to the rectum-sigmoid junction that cross the muscular layer and affect less than one-third of the circumference of the involved intestinal tract. This technique uses a circular or linear stapler introduced transanally, which allows the excision of a full-thickness patch of the rectal wall followed by closure with tightly stapled sutures (53). This technique allows for removal of localized endometriosis nodules and reduces postoperative infectious complications. The bowel is never opened during this procedure. Another alternative approach was introduced by Roman et al. (54), with the Rouen technique that utilized the Contour Transtar stapler (Ethicon Endosurgery) for treatment of large DIE nodules (5-6 cm diameter) that infiltrated the low and mid-rectum. They reported a rectovaginal fistula rate of 7.2% and bladder dysfunction of 9% two years after they performed the Rouen technique in a series of 111 patients. In this study, the risk of postoperative recurrence was 1.8%.

 Laparoscopic colorectal segmental resection should be reserved for patients with multifocal intestinal lesions or large nodules (>3 cm), or in the presence of stenosis (48, 55). This procedure consists of a segmental bowel resection followed by termino-terminal colorectal anastomosis (side-to-end or end-to-end) performed with a transanal circular stapler and a possible protective ileostomy that is related to the distance of the nodule from the anal sphincter. A temporary colostomy may be suggested for nodules situated <6 cm from the anal verge (55). The most frequent complications of this procedure are leakage followed by rectovaginal fistula, with a reported incidence from major available studies that ranged between 1% and 18%. This wide range was due to the variability of patient characteristics; however, most of all the height of rectal involvement and if, during the procedure, both vagina and rectum are opened (56). In surgery for bowel endometriosis, intestinal denervation is always an issue. Patients who underwent segmental resection reported an improvement in symptoms like dyschezia, but less for problems like constipation (even if the intestinal lumen obstruction had been eradicated) (54). This problem could be caused by proximal sectioning of the inferior mesenteric artery where it is surrounded by autonomic nerve fibres, which cause sympathetic denervation of the rectal stump. Raffaelli et al. (57) showed good results in a prospective cohort study, suggesting resection with mesenteric vascular and nerve-sparing surgery that cut the mesentery near the intestinal wall and preserved arteries and autonomic nerves of the mesenteric plexus.

### Ureteral and bladder endometriosis

DIE can affect the ureter extrinsically (with glandular and stromal tissue inside the adventitia and the adjacent connective tissue) or intrinsically (endometriotic nodule intrusion on the muscle layer and basement membrane, invading the lumen) (58). The surgical procedure for ureteral endometriosis (UE) can be conservative (ureterolysis) or more aggressive (ureteroureterostomy, ureteroneocystostomy, nephrectomy) (59). The best approach is often based on the surgeon’s experience and the severity of the lesion. In theory, extrinsic lesions can be treated with ureterolysis, unlike intrinsic ones, which require removal of the involved segment. In practice, it is difficult to establish the depth of the lesion and the involvement of the ureteral wall before surgery, when the only sign of an intrinsic lesion could be the hydroureter. Soriano et al. (60), in a series of 45 patients with UE, suggested preoperative ureteral stenting in case of hydronephrosis, hydroureter, or abnormal urinary function to reduce the ureteral injury rate during surgery. Bosev et al. (61) and Uccella et al. (62) showed that, in the hands of experienced surgeons, ureterolysis might be performed with a low risk of complications (<1%). During the surgery, the dilemma exists about which level of ureterolysis could be considered sufficient, and how surgeons could predict the recovery of its functionality. Bosev et al. (61) suggested inserting a stent if the ureter should still be dilated after ureterolysis, since the surgeon could consider a resection of the stenotic segment or ureteroneocystostomy if it could not be decompressed. Instead, Soriano et al. (60) recommended a ureteroneocystostomy as a primary procedure in cases of ureteral fibrosis after ureterolysis and especially when the obstruction is <2 cm of the insertion of the bladder, or there is sizeable ureteral stenosis. A higher risk of perioperative complications and recurrences in the presence of large endometriotic nodules (>3 cm) or hydronephrosis grade >2 was demonstrated in a case series by Uccella et al. (62).

Two techniques have been described for surgical treatment of bladder endometriosis, transurethral resection (TUR) and partial cystectomy (segmental bladder resection) (63). During laparoscopic partial cystectomy, the decision to perform ureteral cannulation depends on the position of the endometriotic nodule in the bladder wall and the distance from the interureteric ridge (64). In many studies, partial cystectomy has demonstrated its effectiveness with good long-term outcomes. Fedele et al. (65) showed how this technique could be more effective in terms of symptom recurrence if a 1 cm deep myometrial resection of the anterior uterine wall is added during the procedure to eliminate all the adenomyotic foci that could be under the vesical lesion. A combination of TUR and laparoscopic surgery was described by Pontis et al. (66) with good results. In the case of significant endometriotic lesions, this combination allowed for complete removal of the nodule, sparing the removal of healthy bladder tissue and improving the patient’s quality of life.

## Conclusion

DIE is considered the most aggressive of the three phenotypes that constitute endometriosis because it can affect the whole pelvis, subverting the anatomy and functionality of vital organs, with a profoundly negative impact on the patient’s quality of life. 

Once a diagnosis is determined, medical therapy can control the symptoms and stop the development of pathology, keeping in mind the side effects derived from a longterm treatment and the risk of recurrence once suspended. Surgical treatment should be proposed only when strictly necessary (failed hormone therapy, contraindications to hormone treatment, severity of symptoms, infertility), but a conservative approach performed by a multidisciplinary team is preferred when possible. 

There are no studies in the literature that directly compare medical versus surgical therapy in the treatment of endometriosis. Therefore, superiority of one approach over the other cannot be established.

All therapeutic possibilities have to be explained by the physicians in order to help the women make the right choice and minimize the impact of this disease on their lives. 

As for future prospects, the goals of surgery are to make current techniques as conservative as possible towards the function, and radical towards the disease. Instead, medical therapy is focusing on new discoveries in the field of neuroendocrinology and genomics.

## References

[1] Angioni S (2017). New insights on endometriosis. Minerva Ginecol.

[2] Eisenberg VH, Weil C, Chodick G, Shalev V (2018). Epidemiology of endometriosis: a large population-based database study from a healthcare provider with 2 million members. BJOG.

[3] Alio L, Angioni S, Arena S, Bartiromo L, Bergamini V, Berlanda N (2019). Endometriosis: seeking optimal management in women approaching menopause. Climacteric.

[4] Vitale SG, La Rosa VL, Rapisarda AMC, Laganà AS (2017). Impact of endometriosis on quality of life and psychological well-being. J Psychosom Obstet Gynaecol.

[5] Šalamun V, Verdenik I, Laganà AS, Vrtačnik-Bokal E (2018). Should we consider integrated approach for endometriosis-associated infertility as gold standard management?. Rationale and results from a large cohort analysis. Arch Gynecol Obstet.

[6] Nisolle M, Donnez J (1997). Peritoneal endometriosis, ovarian endometriosis, and adenomyotic nodules of the rectovaginal septum are three different entities. Fertil Steril.

[7] Koninckx PR, Ussia A, Adamyan LV, Wattiez A, Donnez J (2012). Deep endometriosis: definition, diagnosis, and treatment. Fertil Steril.

[8] Nisenblat V, Bossuyt PM, Farquhar C, Johnson N, Hull ML (2016). Imaging modalities for the non-invasive diagnosis of endometriosis. Cochrane Database Syst Rev.

[9] Deiana D, Gessa S, Anardu M, Daniilidis A, Nappi L, D’Alterio MN (2019). Genetic of endometriosis: a comprehensive review. Gynecol Endocrinol.

[10] Angioni S, D’Alterio MN, Coiana A, Anni F, Gessa S, Deiana D (2020). Genetic characterization of endometriosis patients: review of the literature and a prospective cohort study on a Mediterranean population. Int J Mol Sci.

[11] Lagana AS, Garzon S, Götte M, Viganò P, Franchi M, Ghezzi F (2019). The pathogenesis of endometriosis: molecular and cell biology insights. Int J Mol Sci.

[12] Viganò D, Zara F, Pinto S, Loddo E, Casula L, Soru MB (2020). How is small bowel permeability in endometriosis patients?. A case control pilot study. Gynecol Endocrinol.

[13] Tosti C, Pinzauti S, Santulli P, Chapron C, Petraglia F (2015). Pathogenetic mechanism of deep infiltrating endometriosis. Reprod Sci.

[14] Gargett CE, Schwab KE, Brosens JJ, Puttemans P, Benagiano G, Brosens I (2014). Potential role of endometrial stem/progenitor cells in the pathogenesis of early-onset endometriosis. Mol Hum Reprod.

[15] Laganà AS, Salmeri FM, Vitale SG, Triolo O, Götte M (2018). Stem cell trafficking during endometriosis: may epigenetics play a pivotal role?. Reprod Sci.

[16] De Pereira LB, Braga NP, Mendonca M, Moro L, Geber S (2012). Apoptosis of ectopic endometrial cells is impaired in women with endometriosis. J Endometr Pelvic Pain Disord.

[17] Machado DE, Abrao MS, Berardo PT, Takiya CM, Nasciutti LE (2008). Vascular density and distribution of vascular endothelial growth actor (VEGF) and its receptor VEGFR-2 (Flk-1) are significantly higher in patients with deeply infiltrating endometriosis affecting the rectum. Fertil Steril.

[18] Chapron C, Fauconnier A, Vieira M, Barakat H, Dousset B, Pansini V (2003). Anatomical distribution of deeply infiltrating endometriosis: surgical implications and proposition for a classification. Hum Reprod.

[19] Triolo O, Laganà AS, Sturlese E (2013). Chronic pelvic pain in endometriosis: an overview. J Clin Med Res.

[20] Noventa M, Scioscia M, Schincariol M, Cavallin F, Pontrelli G, Virgilio B (2019). Imaging modalities for diagnosis of deep pelvic endometriosis: comparison between trans-vaginal sonography, rectal endoscopy sonography and magnetic resonance imaging.A headto-head meta-analysis. Diagnostics (Basel).

[21] Faccioli N, Foti G, Manfredi R, Mainardi P, Spoto E, Ruffo G (2010). Evaluation of colonic involvement in endometriosis: doublecontrast barium enema vs.magnetic resonance imaging. Abdom Imaging.

[22] Vercellini P, Viganò P, Buggio L, Somigliana E (2018). We Can Work It Out: The hundred years' war between experts of surgical and medical treatment for symptomatic deep endometriosis. J Minim Invasive Gynecol.

[23] Szubert M, Zietara M, Suzin J (2018). Conservative treatment of deep infiltrating endometriosis: review of existing options. Gynecol Endo crinol.

[24] Parazzini F, Vigano P, Candiani M, Fedele L (2013). Diet and endometriosis risk: a literature review. Reprod Biomed Online.

[25] Stochino Loi E, Pontis A, Cofelice V, Pirarba S, Fais MF, Daniilidis A (2019). Effect of ultramicronized-palmitoylethanolamide and comicronized palmitoylethanolamide/polydatin on chronic pelvic pain and quality of life in endometriosis patients: An open-label pilot study. Int J Womens Health.

[26] Ferrero S, Camerini G, Ragni N, Venturini PL, Biscaldi E, Remorgida V (2010). Norethisterone acetate in the treatment of colorectal endometriosis: a pilot study. Hum Reprod.

[27] Leonardo-Pinto JP, Benetti-Pinto CL, Cursino K, Yela DA (2017). Dienogest and deep infiltrating endometriosis: the remission of symptoms is not related to endometriosis nodule remission. Eur J Obstet Gynecol Reprod Biol.

[28] Yela D, Kajikawa P, Donati L, Cursino K, Giraldo H, Benetti-Pinto CL (2015). Deep infiltrating endometriosis treatment with dienogest: a pilot study. J Endometr Pelvic Pain Disord.

[29] Angioni S, Nappi L, Pontis A, Sedda F, Luisi S, Mais V (2015). Dienogest.A possible conservative approach in bladder endometriosis.Results of a pilot study. Gynecol Endocrinol.

[30] Angioni S, Pontis A, Malune ME, Cela V, Luisi S, Litta P (2020). Is dienogest the best medical treatment for ovarian endometriomas?. Results of a multicentric case control study. Gynecol Endocrinol.

[31] Tarjanne S, Ng CHM, Manconi F, Arola J, Mentula M, Maneck B (2015). Use of hormonal therapy is associated with reduced nerve fiber density in deep infiltrating, rectovaginal endometriosis. Acta Obstet Gynecol Scand.

[32] Casper RF (2017). Progestin-only pills may be a better first-line treatment for endometriosis than combined estrogen-progestin contraceptive pills. Fertil Steril.

[33] Dunselman GAJ, Vermeulen N, Becker C, Calhaz-Jorge C, D’Hooghe T, De Bie B (2014). ESHRE guideline: management of women with endometriosis. Hum Reprod.

[34] Fedele L, Bianchi S, Zanconato G, Tozzi L, Raffaelli R (2000). Gonadotropin releasing hormone agonist treatment for endometriosis of the rectovaginal septum. Am J Obstet Gynecol.

[35] Roman H, Saint-Ghislain M, Milles M, Marty N, Hennetier C, Moatassim S (2015). Improvement of digestive complaints in women with severe colorectal endometriosis benefiting from continuous amenorrhoea triggered by triptoreline.A prospective pilot study. Gynecol Obstet Fertil.

[36] Angioni S, Pontis A, Dessole M, Surico D, De Cicco Nardone C, Melis I (2015). Pain control and quality of life after laparoscopic en-block resection of deep infiltrating endometriosis (DIE) vs.incomplete surgical treatment with or without GnRHa administration after surgery. Arch Gynecol Obstet.

[37] Melis GB, Neri M, Corda V, Malune ME, Piras B, Pirarba S (2016). Overview of elagolix for the treatment of endometriosis. Expert Opin Drug Metab Toxicol.

[38] Selak V, Farquhar C, Prentice A, Singla A (2007). Danazol for pelvic pain associated with endometriosis. Cochrane Database Syst Rev.

[39] Razzi S, Luisi S, Calonaci F, Altomare A, Bocchi C, Petraglia F (2007). Efficay of vaginal danazol treatment in women with recurrent deeply infiltrating endometriosis. Fertil Steril.

[40] Patwardhan S, Nawathe A, Yates D, Harrison GR, Khan KS (2008). Systematic review of the effects of aromatase inhibitors on pain associated with endometriosis. BJOG.

[41] Ferrero S, Venturini PL, Gillott DJ, Remorgida V (2011). Letrozole and norethisterone acetate versus letrozole and triptorelin in the treatment of endometriosis related pain symptoms: a randomized controlled trial. Reprod Biol Endocrinol.

[42] Bedaiwy MA, Alfaraj S, Yong P, Casper R (2017). New developments in the medical treatment of endometriosis. Fertil Steril.

[43] Kulak J Jr, Fischer C, Komm B, Taylor HS (2011). Treatment with bazedoxifene, a selective estrogen receptor modulator, causes regression of endometriosis in a mouse model. Endocrinology.

[44] Laganà AS, La Rosa VL (2020). Multidisciplinary management of endometriosis: current strategies and future challenges. Minerva Med.

[45] Ceccaroni M, Clarizia R, Roviglione G, Ruffo G (2013). Neuro-anatomy of the posterior parametrium and surgical considerations for a nervesparing approach in radical pelvic surgery. Surg Endosc.

[46] Ceccaroni M, Clarizia R, Bruni F, D’Urso E, Gagliardi ML, Roviglione G (2012). Nerve-sparing laparoscopic eradication of deep endometriosis with segmental rectal and parametrial resection: the Negrar method.A single-center, prospective, clinical trial. Surg Endosc.

[47] Angioni S, Peiretti M, Zirone M, Palomba M, Mais B, Gomel V (2006). Laparoscopic excision of posterior vaginal fornix in the treatment of patients with deep endometriosis without rectum involvement: surgical treatment and long-term follow-up. Hum Reprod.

[48] Donnez O, Roman H (2017). Choosing the right surgical technique for deep endometriosis: shaving, disc excision, or bowel resection?. Fertil Steril.

[49] Laganà AS, Vitale SG, Trovato MA, Palmara VI, Rapisarda AMC, Granese R (2016). Full-thickness excision versus shaving by laparoscopy for intestinal deep infiltrating endometriosis: rationale and potential treatment options. Biomed Res Int.

[50] Kondo W, Bourdel N, Tamburro S, Cavoli D, Jardon K, Rabishong B (2011). Complications after surgery for deeply infiltrating pelvic endometriosis. BJOG.

[51] Roman H, Milles M, Vassilieff M, Resh B, Tuech JJ, Huet E (2016). Long-term functional outcomes following colorectal resection versus shaving for rectal endometriosis. Am J Obstet Gynecol.

[52] Meuleman C, Tomassetti C, D’Hoore A, Cleynenbreugel BV, Penninckx F, Vergote I (2011). Surgical treatment of deeply infiltrating endometriosis with colorectal involvment. Hum Reprod Update.

[53] Gordon SJ, Mather PJ, Woods R (2001). Use of the CEEA stapler to avoid ultra-low segmental resection of a full-thickness rectal endometriotic nodule. J Am Assoc Gynecol Laparosc.

[54] Roman H, Darwish B, Bridoux V, Chati R, Kermiche S, Coget J (2017). Functional outcomes after disc excision in deep endometriosis of the rectum using transanal staplers: a series of 111 consecutive patients. Fertil Steril.

[55] Abrao MS, Borrelli GM, Clarizia R, Kho RM, Ceccaroni M (2017). Strategies for management of colorectal endometriosis. Semin Reprod Med.

[56] Tarjanne S, Heikinheimo O, Mentula M, Härkki P (2015). Complications and long-term follow-up on colorectal resections in the treatment of deep infiltrating endometriosis extending to bowel wall. Acta Obstet Gynecol Scand.

[57] Raffaelli R, Garzon S, Baggio S, Genna M, Pomini P, Laganà AS (2018). Mesenteric vascular and nerve sparing surgery in laparoscopic segmental intestinal resection for deep infiltrating endometriosis. Eur J Obstet Gynecol Reprod Biol.

[58] Chapron C, Chiodo I, Leconte M, Amsellem-Ouazana D, Chopin N, Borghese B (2010). Severe ureteral endometriosis: the intrinsic type is not so rare after complete surgical exeresis of deep endometriotic lesions. Fertil Steril.

[59] Butticè S, Laganà AS, Mucciardi G, Marson F, Tefik T, Netsch C (2016). Different patterns of pelvic ureteral endometriosis.What is the best treatment? Results of a retrospective analysis. Arch Ital Urol Androl.

[60] Soriano D, Schonman R, Nadu A, Lebovitz O, Schiff E, Seidman DS (2011). Multidisciplinary team approach to management of severe endometriosis affecting the ureter: long-term outcome data and treatment algorithm. J Minim Invasive Gynecol.

[61] Bosev D, Nicoll LM, Bhagan L, Lemyre M, Payne C, Gill H (2009). Laparoscopic management of ureteral endometriosis: the Stanford University Hospital experience with 96 consecutive cases. J Urol.

[62] Uccella S, Cromi A, Casarin J, Bogani G, Pinelli C, Serati M (2014). Laparoscopy for ureteral endometriosis: surgical details, long-term follow-up, and fertility outcomes. Fertil Steril.

[63] Saccardi C, Vitagliano A, Litta P (2017). Bladder endometriosis: a summary of current evidence. Minerva Ginecol.

[64] Seracchioli R, Mabrouk M, Montanari G, Manuzzi L, Concetti S, Venturoli S (2010). Conservative laparoscopic management of urinary tract endometriosis (UTE): surgical outcome and long-term followup. Fertil Steril.

[65] Fedele L, Bianchi S, Zanconato G, Bergamini V, Berlanda N, Carmignani L (2005). Long-term follow-up after conservative surgery for bladder endometriosis. Fertil Steril.

[66] Pontis A, Nappi L, Sedda F, Multinu F, Litta P, Angioni S (2016). Management of bladder endometriosis with combined transurethral and laparoscopic approach.Follow-up of pain control, quality of life, and sexual function at 12 months after surgery. Clin Exp Obstet Gynecol.

